# A systematic review on poly(I:C) and poly-ICLC in glioblastoma: adjuvants coordinating the unlocking of immunotherapy

**DOI:** 10.1186/s13046-021-02017-2

**Published:** 2021-06-25

**Authors:** Jorrit De Waele, Tias Verhezen, Sanne van der Heijden, Zwi N. Berneman, Marc Peeters, Filip Lardon, An Wouters, Evelien L. J. M. Smits

**Affiliations:** 1 Center for Oncological Research (CORE), Integrated Personalized & Precision Oncology Network (IPPON), University of Antwerp, Universiteitsplein 1, B-2610 Antwerp, Belgium; 2 Laboratory of Experimental Hematology, University of Antwerp, Universiteitsplein 1, B-2610 Antwerp, Belgium; 3 Department of Hematology, Antwerp University Hospital, Wilrijkstraat 10, B-2650 Edegem, Belgium; 4 Center for Cell Therapy and Regenerative Medicine, Antwerp University Hospital, Wilrijkstraat 10, B-2650 Edegem, Belgium; 5 Multidisciplinary Oncological Center Antwerp, Antwerp University Hospital, Wilrijkstraat 10, B-2650 Edegem, Belgium

**Keywords:** Poly(I:C), Poly-ICLC (Hiltonol), Glioblastoma, Immunotherapy, Immune checkpoint, Vaccination, Combination therapy, Toll-like receptor 3, Adjuvant, Glioma

## Abstract

Immunotherapy is currently under intensive investigation as a potential breakthrough treatment option for glioblastoma. Given the anatomical and immunological complexities surrounding glioblastoma, lymphocytes that infiltrate the brain to develop durable immunity with memory will be key. Polyinosinic:polycytidylic acid, or poly(I:C), and its derivative poly-ICLC could serve as a priming or boosting therapy to unleash lymphocytes and other factors in the (immuno)therapeutic armory against glioblastoma. Here, we present a systematic review on the effects and efficacy of poly(I:C)/poly-ICLC for glioblastoma treatment, ranging from preclinical work on cellular and murine glioblastoma models to reported and ongoing clinical studies. MEDLINE was searched until 15 May 2021 to identify preclinical (glioblastoma cells, murine models) and clinical studies that investigated poly(I:C) or poly-ICLC in glioblastoma. A systematic review approach was conducted according to PRISMA guidelines. ClinicalTrials.gov was queried for ongoing clinical studies. Direct pro-tumorigenic effects of poly(I:C) on glioblastoma cells have not been described. On the contrary, poly(I:C) changes the immunological profile of glioblastoma cells and can also kill them directly. In murine glioblastoma models, poly(I:C) has shown therapeutic relevance as an adjuvant therapy to several treatment modalities, including vaccination and immune checkpoint blockade. Clinically, mostly as an adjuvant to dendritic cell or peptide vaccines, poly-ICLC has been demonstrated to be safe and capable of eliciting immunological activity to boost therapeutic responses. Poly-ICLC could be a valuable tool to enhance immunotherapeutic approaches for glioblastoma. We conclude by proposing several promising combination strategies that might advance glioblastoma immunotherapy and discuss key pre-clinical aspects to improve clinical translation.

## Background

Glioblastoma (GBM) is the most common primary brain tumor and remains one of the deadliest cancers to date. The contemporary first-line multimodal standard of care (SOC), as defined by the landmark 2005 EORTC TMZ trial [1], consists of maximal surgical resection followed by chemoradiation and adjuvant temozolomide (TMZ), but it only renders a 14.6-month median overall survival (mOS) and a 7.2% five-year survival [1, 2]. Major therapeutic hurdles include incomplete surgical resection, due to the invading nature of GBM, and therapeutic resistance, due to refractory cells in the heterogenous GBM cell population. Inevitably, recurrence occurs, at which point a patient’s only option are salvage treatment modalities, which are often experimental with limited efficacy [3]. With the current SOC having “celebrated” its 15^th^ anniversary last year, it is evident that the therapeutic landscape for GBM is unquestionably in dire need of rejuvenation with novel and effective treatment options.

Immunotherapy for GBM treatment is intensively under investigation as the immune system theoretically possesses proficient features against the heterogeneous nature of GBM. However, GBM has adapted an immunosuppressive state which will have to be conquered [4]. The aim of immunotherapy is to enable immune cells to recognize and eradicate refractory GBM cells, while developing memory to prevent tumor recurrence. Multiple avenues to achieve this are being pursued, including dendritic cell (DC) vaccination, immune checkpoint blockade (ICB), chimeric antigen receptor (CAR) T cells and oncolytic viruses [4]. In this review, we focused on polyinosinic:polycytidylic acid or poly(I:C), and its derivative stabilized with carboxymethylcellulose and poly-L-lysine (poly-ICLC), adjuvants included on the Cancer Immunotherapy Trials Network’s priority list of agents to boost cancer immunotherapy. Both poly(I:C) and poly-ICLC hold great potential to becoming pivotal, adjuvant components in the multifaceted immunotherapy approach to GBM.

**Fig. 1:**
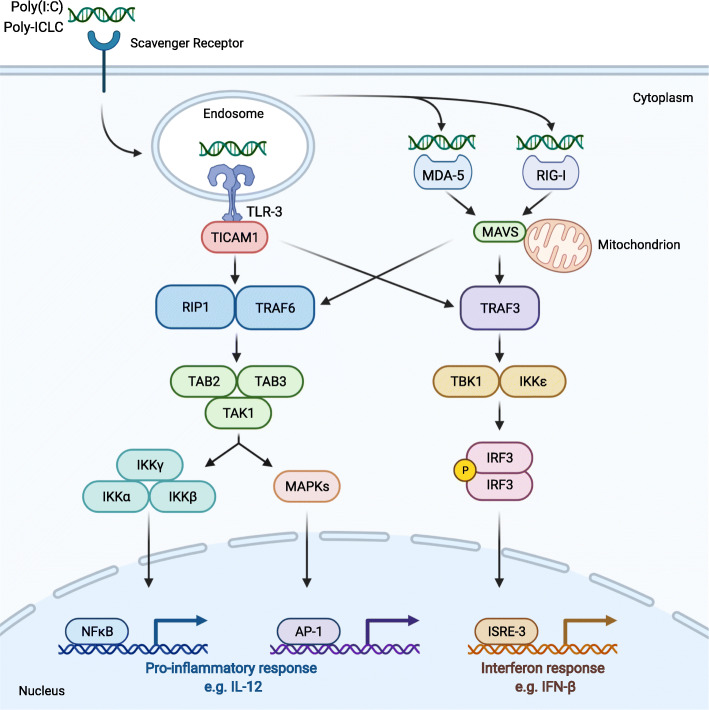
Poly(I:C) and poly-ICLC signaling through TLR-3, MDA-5 and RIG-I generates a pro-inflammatory and interferon response. Poly(I:C) and poly-ICLC bind either the endosomal receptor TLR-3, leading to recruitment of adaptor molecule TICAM1, or the cytoplasmic receptors MDA-5 or RIG-I, which signal through the mitochondrial MAVS adaptor protein. Downstream both TICAM1 and MAVS transduce similar signal pathways via TRAF3 and TRAF6, though with distinct emphasis. TRAF3 leads to TBK1-IKKε complex formation, which results in phosphorylation of an IRF3 dimer that will induce an IFN response via IRSE3. TRAF6, along with RIP1, complexes TAK1 with TAB2 and TAB3, activating MAPK to activate transcription factor AP-1. The TAK1 complex will also activates the IKKα/β/γ complex, allowing transcription of NFκB. Both AP-1 and NFκB results in pro-inflammatory cytokines and chemokines. Hence, poly(I:C) and poly-ICLC signaling result in an immunostimulatory response. AP-1, activator protein 1; IFN, interferon; IKKα/β/γ, inhibitor of NFκB kinase regulatory subunit α/β/γ; IL-12, interleukin-12; IRF3, IFN regulatory factor 3; ISRE-3, IFN-stimulated response element 3; MAPKs, mitogen-activated protein kinases; MAVS, mitochondrial antiviral signaling protein; MDA-5, melanoma differentiation-associated gene 5; NFκB, nuclear factor κ-light-chain-enhancer of activated B cells; Poly(I:C), polyinosinic:polycytidylic acid; Poly-ICLC, poly(I:C) stabilized with carboxymethylcellulose and poly-L-lysine; RIG-I, retinoic acid-inducible gene I; RIP1, receptor-interacting serine/threonine-protein kinase 1; TAB, TAK1-binding protein; TAK1, transforming growth factor β activated kinase 1; TBK1, TRAF family member associated NFκB activator binding kinase 1; TICAM1, TLR adaptor molecule 1; TLR-3, Toll-like receptor 3; TRAF; tumor necrosis factor receptor associated factor 3

Poly(I:C) and poly-ICLC are synthetic double-stranded RNA molecules (dsRNA) consisting of a polyinosinic acid homopolymer annealed to a polycytidylic acid homopolymer, thus resulting in a stable double helix [5]. They bind to endosomal Toll-like receptor (TLR)-3 and the cytoplasmic receptors retinoic acid-inducible gene I (RIG-I) and melanoma differentiation-associated gene 5 (MDA-5) [6], as shown in Fig. 1. As such, they mimic a viral infection, consequently eliciting the secretion of type I interferon (IFN) and pro-inflammatory cytokines, which play prominent roles in inducing an immune response [6]. TLR-3 and MDA-5/RIG-I both elicit their signaling function via recruitment of an adaptor protein, TLR adaptor molecule 1 (TICAM1) and the mitochondrial antiviral signaling protein (MAVS), respectively. Both pathways converge to tumor necrosis factor (TNF) receptor-associated factors TRAF3 and TRAF6. TRAF3 leads to complexation of TBK1, TRAF family member-associated nuclear factor κ-light-chain-enhancer of activated B cells (NFκB) activator binding kinase 1, and inhibitor of NFκB kinase subunit ε (IKKε). This complex phosphorylates IFN regulatory factor 3 (IRF3) dimer, which then binds onto IFN-stimulated response element 3 (IRSE-3) following nuclear translocation. This leads to transcription of type I IFN, in particular IFN-β, as well as genes coding for lymphocyte trafficking molecules. TRAF6, in concert with receptor-interacting serine/threonine-protein kinase 1 (RIP1), leads to a transforming growth factor (TGF)-β activated kinase 1 (TAK1) complex that phosphorylates two downstream pathways. On the one hand, the IKKα/β/γ complex relieves NFκB from its suppressor NFκB inhibitor α, allowing nuclear translocation. On the other hand, phosphorylation of mitogen-activated protein kinases (MAPK) leads to activation of activator protein 1 (AP-1). Both NFκB and AP-1 generate pro-inflammatory cytokines and chemokines [6–8]. Although the endosomal and cytoplasmic receptors signal through similar pathways, some distinctions exist. While TLR-3 activation favors pro-inflammatory cytokines, the endosomal receptors are more inclined to produce type I IFN, with IRF7 activation more readily in the picture, and interleukin (IL)-15 [9]. Also the formulation of dsRNA influences the outcome. Poly(I:C) more activates TLR-3, with the cytoplasmic receptors being activated following endosomal leakage and bearing a preference for high (MDA-5) or low molecular weight (RIG-I) poly(I:C) [10, 11]. Poly-ICLC, by virtue of the components that stabilize poly(I:C), results in stronger activation of cytoplasmic receptor signaling by virtue of endosomal rupture due to the components to stabilize poly(I:C) [9, 11].

**Fig. 2:**
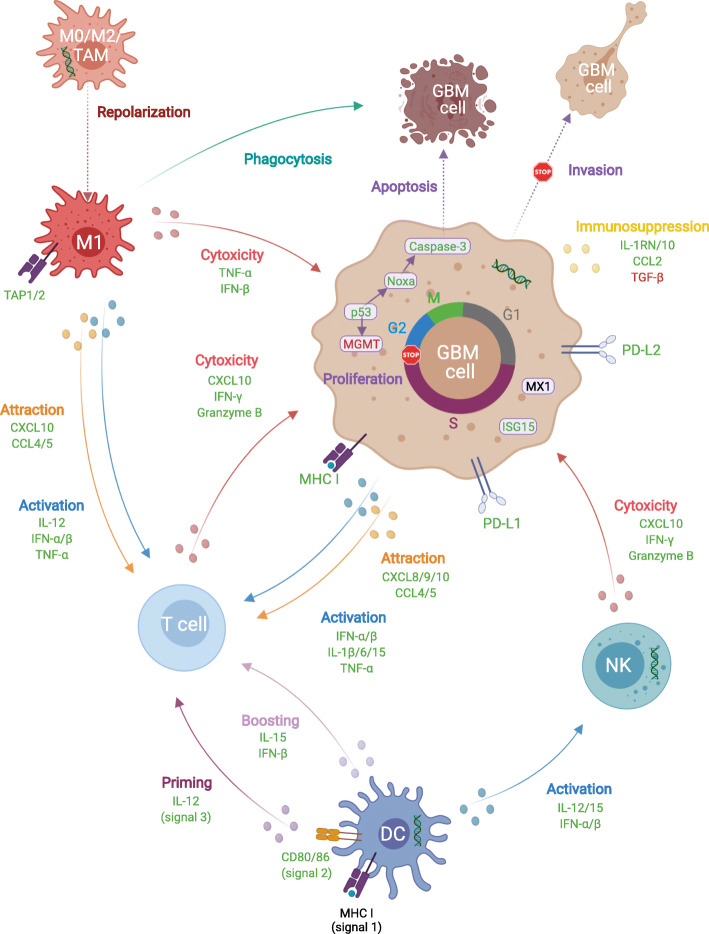
Integrated overview on how poly(I:C) affects GBM and immune cells on molecular and functional levels. Poly(I:C) activates several immune cells directly and indirectly, while also leveraging the GBM cellular machinery for attraction and activation of immune cells. In addition, poly(I:C) can induce cytostasis, apoptosis and less invasiveness, while remaining sensitivity to certain viruses. Color coding of proteins indicates effect of poly(I:C) compared to non-treated cells: black, unaltered; green, upregulation/induction; red, downregulation/inhibition. Green helices represent double-stranded poly(I:C); small spheres represent secreted factors. CCL/CXCL, C-C/C-X-C motif chemokine ligand; GBM, glioblastoma; IFN, interferon; IL, interleukin; IL-1RN, IL-1 receptor antagonist; ISG15, IFN-stimulated gene 15; M0/1/2, M0/1/2-polarized macrophage; MGMT, O^6^-methylguanine-DNA methyltransferase; MHC, major histocompatibility complex; MX1, MC dynamin-line GTPase 1; NK, natural killer cell; Noxa, phorbol-12-myristate-13-acetate-induced protein 1; PD-L, programmed death 1 ligand; poly(I:C), polyinosinic:polycytidylic acid; TGF, transforming growth factor; TAM, tumor-associated macrophage; TAP1/2, transporter 1/2, ATP binding cassette subfamily B member; TNF, tumor necrosis factor

Due to its capacity to activate many immune cell types directly and indirectly, as depicted in Fig. 2, poly(I:C) is distinctly known for its immunostimulatory activity [12]. In this context, it is primarily renowned as a priming agent for activating antigen-presenting cells, in particular dendritic cells (DC) [13]. Indeed, poly(I:C) activates DC to strongly upregulate signals required for antigen-specific T-cell priming, as depicted in Fig. 2, which include: co-stimulatory molecules CD80, CD86 (signal 2) and CD40; pro-inflammatory cytokines such as IL-12 (signal 3); and, chemokines that attract T cells, e.g. CXCL10 [13–15]. Furthermore, poly(I:C) treatment of DC in a booster phase stimulates secondary T-cell expansion to a much greater extent than other TLR agonists, through type I IFN and IL-15 signaling [9, 16]. Therefore, poly(I:C) has regularly been used in DC maturation protocols in vitro in order to obtain efficient DC products, outperforming TLR-4 agonist LPS and a pro-inflammatory cytokine cocktail [13]. Moreover, in mice, poly(I:C) is the most effective inducer of type I IFN among TLR agonists [17]. In addition, poly(I:C)-treated DC activate natural killer (NK) cells through both IL-12 secretion and cell-cell contact [18]. Direct activation of NK cells by poly(I:C) has been reported as well, although reported data is conflicting [19, 20]. Next to DC, poly(I:C) also profoundly affects macrophages, in a similar way. Interestingly, while it has been reported to repolarize M2 and tumor-associated macrophages (TAM) with tumoricidal and enhanced phagocytic capacities, it can also attract and stimulate T cells [21–23]. In addition to its effects on immune cells, poly(I:C) also affects other cells in the tumor microenvironment (TME). It has been shown to directly inhibit tumor growth via suppression of proliferation and induction of apoptosis in several tumor types [12]. Indeed, its viral mimicry can activate pathways to protect neighboring cells from infection, potentially culminating in cellular suicide [24]. Therefore, poly(I:C) is generally seen as a cytotoxic agent for tumor cells, but few studies reporting poly(I:C)-mediated stimulation of cancer cell migration, urge caution in its use [25, 26]. Indeed, invasion of GBM cells into healthy parenchyma impedes surgical resection and paves the way for tumor recurrence. Therefore, poly(I:C) must be studied in the context of each tumor type. Here, we conducted a systematic review to evaluate the state-of-the-art regarding the effects of poly(I:C) and poly-ICLC in GBM in order to evaluate its potential to boost immunotherapy for GBM. First, we examined in vitro studies to provide an overview of the direct effects of poly(I:C) on GBM cells. Next, we assessed pre-clinical in vivo studies, which also take into account the direct and indirect actions of poly(I:C) on neighboring stromal and peripheral immune cells. Afterwards, we described clinical experiences with poly-ICLC in GBM while looking ahead to trials currently ongoing. Finally, we concluded with a discussion on its position in the medical landscape for GBM and postulate future perspectives.

## Methods

**Fig. 3:**
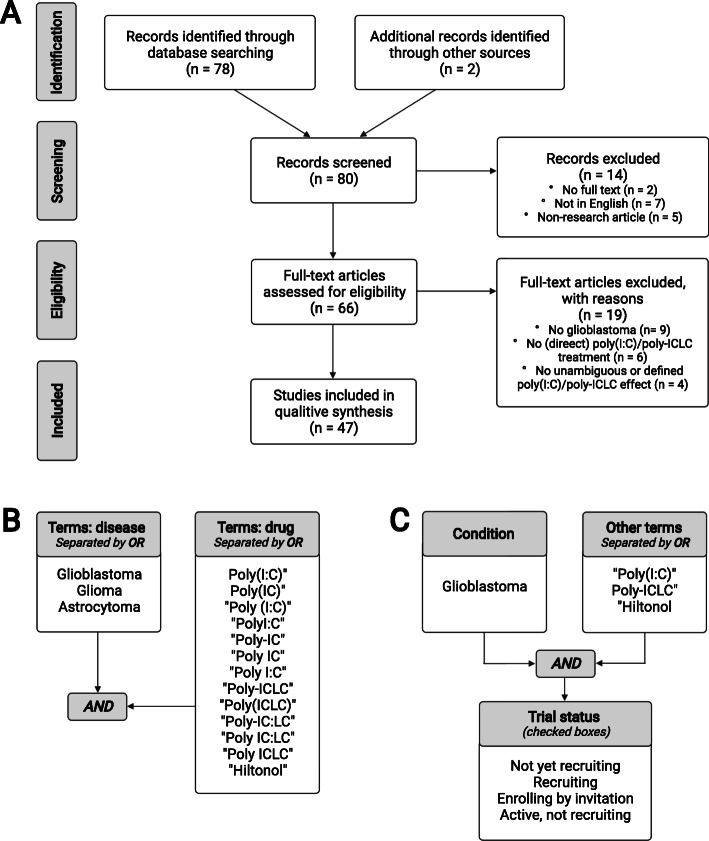
Search strategies and assessment pipeline of systematic search. (**A**) PRISMA flow hart depicting the systematic assessment. (**B**-**C**) Search term applied to MEDLINE and clinicaltrials.gov, respectively. AND and OR represent Boolean operators

We executed a systematic review according to the Preferred Reporting Items for Systematic Review and Meta-Analyses (PRISMA) guidelines [27], as depicted in Fig. 3A. The search was conducted in the MEDLINE database (1973-present) using the terms displayed in Fig. 3B, ending on 15 May 2021. Research articles with data on poly(I:C) or poly-ICLC in GBM were included provided a full-text written in English was available. Reviews and perspectives were excluded, as well as articles that (i) did not use poly(I:C)/poly-ICLC, (ii) did not utilize GBM as target tumor model, (iii) presented non-specified or ambiguously-described in vitro effects of poly(I:C)/poly-ICLC, (iv) did not administer poly(I:C)/poly-ICLC directly to test objects in studies in vivo, or (v) only reported on poly(I:C)/poly-ICLC-induced cellular signaling. We did not include other poly(I:C) derivatives, Ampligen (latest report in 1991) and BO-112 (novel and not yet tested in GBM). In addition, we searched for ongoing clinical trials with poly-ICLC on clinicaltrials.gov, using the strategy presented in Fig. 3C, which ended on 27 December 2020.

### In vitro effect of poly(I:C) on GBM cells

Since poly(I:C) is primarily known in an immunological context, we first discuss the observations regarding interactions of GBM cells with the immune system following poly(I:C) treatment. Cell health as a direct target of poly(I:C) is subsequently discussed. Figure 2 presents an integrated response of GBM and immune cells to poly(I:C) and resulting (inter)cellular effects.

#### Immunomodulation

Although predominantly associated with poly(I:C)-mediated immune cell activation, poly(I:C)/poly-ICLC also induces type I IFN gene transcription and subsequent secretion of IFN-α and IFN-β by both human and murine GBM cells [28–40]. Surprisingly, poly(I:C) did not initiate IFN-β transcription in C6 rat glioma cells and some human GBM cell lines [41, 42]. In contrast, primary human GBM (pGBM) cells induced much stronger IFN-β secretion compared to IFN-α, while type II IFN-γ secretion was absent [30]. Of note, two reports showed a stronger IFN-β secretion upon targeting poly(I:C) to its cytosolic receptors rather than to TLR-3 [28, 31], suggesting that the robustness of the poly(I:C)-induced type I IFN response might depend on which receptor pathway is primarily targeted. Nonetheless, promoting endocytosis in murine GBM cells also elevated IFN-β secretion [40]. Besides type I IFN, poly(I:C) also characteristically induces a release of pro-inflammatory cytokines [6]. This has also been the case in GBM cells, as evidenced by upregulated TNF-α, IL-1β, IL-6 and IL-15 [29, 30, 43–46]. Interestingly, poly(I:C) also downregulates the secretion of the strongly immunosuppressive TGF-β [30], although it did increase the release of IL-1R antagonist and IL-10 [23]. A poly(I:C)-induced secretome favoring immunostimulation could lead to immune activation. Indeed, two studies reported immune activation by poly(I:C)-treated pGBM cells. Glas et al. observed a modest increase in type I IFN-dependent degranulation and cytolysis by NK cells, when cocultured with primary GBM stem cells (pGSC) treated with poly-ICLC or transfected poly(I:C) [31]. On the other hand, we demonstrated a significant increase of activated lymphocytes, releasing IFN-γ and granzyme B, when they were cocultured with untransfected poly(I:C)-treated pGBM cells [30].

Similar to the induction of pro-inflammatory cytokines, poly(I:C) elicits the secretion of chemokines from GBM cells which can attract tumorlytic lymphocytes. Poly(I:C)/poly-ICLC, targeted to either receptor pathway, stimulates GBM cell secretion of CXCL8, CXCL9, CXCL10, CCL2, CCL4 and CCL5 [29–34, 37–40, 45]. In transwell assays using supernatant of poly(I:C)-treated pGBM, chemoattraction of CD8^+^ and CD4^+^ T cells, but not NK cells, was increased in a manner that suggested involvement of those ligands for CCR5 and CXCR3 [30].

While cytokines can signal from afar, interactions between membrane proteins on GBM and immune cells are central in dictating immunological activity. For T cells in particular, antigen presentation via major histocompatibility complex (MHC) molecules is crucial in their antigen-specific adaptive capacity [47]. Poly(I:C)/poly-ICLC treatment of human and murine GBM upregulates MHC class I molecules, partly via treatment-induced IFN-β [39, 44, 48]. This suggests that poly(I:C)-treated GBM cells bear enhanced epitope-presenting capacity for infiltrating T cells. On the other hand, MHC class I molecules can also signal inhibition of NK cells [49]. Nonetheless, it has been shown that NK cells become activated by poly(I:C)-treated pGBM cells and exert their lytic function [30, 31], indicating that other factors tip the balance towards antitumoral activity.

In the last decade, there has been an impetus on immunotherapy given the clinical responses achieved by ICB in multiple cancer types [50]. Interestingly, low levels of programmed cell death 1 ligand (PD-L)1 and PD-L2 expression on pGBM cells increase robustly, following untransfected poly(I:C) treatment, partly via downstream IFN-β [30]. Although elevated immune checkpoint ligand expression would impair PD-1^+^ activated lymphocytes, immune activation was observed in cocultures with poly(I:C)-treated pGBM cells [30]. However, additional blockade of PD-L1 further propagated this immune activation. Intriguingly, such enhancement was not detected upon blocking PD-L2, suggesting that this ligand is redundant in GBM-mediated inhibition of immune cell activation [30]. This underscores the potential of targeting these negative feedback counter mechanisms to further invigorate anti-tumor immunity. Moreover, heightened PD-L1/PD-L2 expression is expected with immune activation due to immune cell-secreted IFN-γ [30].

In summary, poly(I:C) - either untransfected, transfected, nanoplexed or in its stabilized form - induces secretion of pro-inflammatory cytokines and chemokines by pGBM cells, which are capable of eliciting in vitro immune attraction and activation. In addition, it demonstrates potency to prime GBM for T-cell immunity when combined with PD-1/PD-L1 blockade; NK cells could also play a role despite the presence of inhibitory signals.

#### Viral sensitivity

Oncolytic viruses are actively being evaluated in clinical trials for GBM (Clinicaltrial.gov: fifteen active and seven completed trials), with first results pointing towards a favorable safety profile and immunogenic activity [51]. While poly(I:C) did not affect the susceptibility of U87 cells, it protected normal human brain cells from viral infection, replication and induced cell death [42, 52]. Protection against VSV-rp30a is possibly conferred by MX1, a cellular antiviral protein that is profoundly induced by poly(I:C) in normal brain cells but barely modulated in GBM cells [40, 42]. These observations argue for the adjuvant use of poly(I:C) in oncolytic virotherapy in order to protect healthy brain cells, though protection should be established upfront. Indeed, poly(I:C) also upregulates ISG15 in both normal brain cells and GBM cells, arguing against the use of e.g. Sindbis virus [42]. In addition, alternative formulations of poly(I:C) might induce MX1 in GBM cells [40], and therefore, the formulation is also of importance in this context.

#### Cell health and behavior

Central to cellular health are viability and proliferation as primary targets in cancer treatment. Poly(I:C)/poly-ICLC affects human GBM cell lines differently with regard to suppressing their growth, be it due to cytostatic or cytotoxic activities [28, 31, 42, 53–55]. Notably, no study observed stimulation of GBM cell proliferation. Discrepant findings were reported regarding poly(I:C) formulations most effective at mediating cytostasis/cytotoxicity. In an early study, the growth inhibitory effect was stronger for untransfected poly(I:C) in comparison to poly(I:C) entrapped in cationic liposomes [28]. In contrast, a more recent study demonstrated that transfected reduced the metabolic activity of (p)GBM cells while untransfected poly(I:C) did not. This was associated with induction of apoptosis through caspase-3 and Noxa and mediated through MDA-5/RIG-I rather than TLR-3 [31]. Moreover, both pGBM/pGSC could be targeted with transfected poly(I:C), whereas several non-malignant brain cell types remained unharmed [31]. Although upregulation of Noxa also occurred in these normal cells, its effect was presumably disarmed by the higher expression of the anti-apoptotic Bcl-2 [31]. Other studies found that transfection of pegylated poly(I:C) rapidly induced apoptosis, further enhanced by modifications that augment cytosolic release [54, 55].

In C6 rat glioma cells, poly(I:C) inhibits ligands, receptors and binding proteins of the insulin-like growth factor system, allowing poly(I:C)-induced IFN-α to mediate cytostasis [41, 56, 57]. Indeed, despite increased DNA synthesis and S phase being increased, a proliferative reduction was achieved due to an S/G2-driven cell cycle arrest. Cytotoxicity was only observed upon long-term culture in starved conditions [41].

Invasion of GBM in the surrounding healthy brain parenchyma presents a paramount problem to achieving complete resection without compromising neurological or motor functions of the patient. Treatment with poly(I:C) diminished invasion of human GBM spheroids embedded in a collagen matrix [22]. Although this effect was not observed with rat GBM spheroids, the absence of an enhanced invasive capacity is particularly important.

In conclusion, there are no indications that poly(I:C) mediates protumoral effects regarding GBM cell health and behavior. In contrast, it has proven to be cytostatic/cytotoxic and potentially anti-invasive, but optimization of its formulation appears to be required to unlock its full potential.

### Effects of poly(I:C) in GBM animal models

In particular for GBM, the existence of the blood-brain-barrier (BBB) imposes a unique complexity. Whereas we focused on the direct effects of poly(I:C)/poly-ICLC on GBM cells in the above chapter, here we elaborate on its general effect in animal GBM models.

#### Safety

To date, none of the preclinical in vivo GBM studies employing poly(I:C) have reported any adverse events (AE) or pathological defects regarding physical, behavioral or neurological characteristics; this includes both in immediate circumstances and in long-term surviving mice [28, 39, 44, 55, 58]. In addition, reports on exacerbation of GBM growth or invasiveness are absent. Hence, no observations indicate any concern for safety.

#### Poly(I:C) as stand-alone treatment

Intratumoral release of epidermal growth factor receptor-targeted pegylated (PEG) poly(I:C) via osmotic micropumps significantly extended the lives of U87 xenograft-implanted mice. Enhanced cytosolic release of PEG-poly(I:C) even completely eradicated large tumors [55]. In addition, bystander effects also killed unsusceptible cells in a mixed xenograft model, suggesting its applicability in heterogeneous tumors such as GBM [55].

In contrast, single-agent treatment with poly(I:C)/poly-ICLC failed to show a robust therapeutic effect in syngeneic CT-2A and GL261 models [39, 44, 58–60], even following tumor-targeted delivery [40], although surviving mice developed immune memory [59]. Nonetheless, poly(I:C) effectively enhanced activation of resident DC in the brain TME, while the number of non-migratory and migratory DC in the tumor-draining lymph nodes (dLN) decreased and increased, respectively. Furthermore, enhanced division of dLN-residing antigen-specific T cells elevated the percentage of TNF-α^+^ antigen-specific T cells in the brain [59], although tumor-infiltrating lymphocytes (TIL) remained scarce [40]. These observations in the brain and dLN suggest that poly(I:C) strengthens the potential for antigen presentation and development of an immune response, which notwithstanding remains largely incapacitated. For instance, poly(I:C) increased PD-L1 expression in the brain and on myeloid cells in dLN and spleen [40, 59]. Hence, these observations advocate for its use in immunotherapeutic combination regimens. In addition, the ability of poly(I:C)/poly-ICLC to produce a pro-inflammatory response was replicated in vivo across the BBB as demonstrated by the transient increase in serum and brain of TNF-α and IFN-α, despite systemic administration [39, 58].

#### Immune checkpoint blockade

As it stands, poly(I:C) single-agent treatment appears to prime both tumor and immune microenvironments for antitumor immunity but is unable to single-handedly unleash it. In this regard, GBM cells and DC have been shown to counter the poly(I:C)-driven pro-inflammatory signature by upregulating immune checkpoint ligands PD-L1 and PD-L2 [30, 40, 59]. Consequently, poly(I:C) and anti-PD-1 therapy synergize to provide a great survival benefit including immune memory in the GL261 in vivo model [59]. This was accompanied by decreased myeloid and regulatory T cells (Treg) in the brain, which welcomed more non-migratory DC and IFN-γ^+^CD8^+^ T effector cells. In the dLN, more activated myeloid and memory T (Tmem) cells were observed. The therapeutic synergy was fully dependent on cross-presenting DC, demonstrating that this benefit was related to the activation of antigen presentation, the priming and expanding of antigen-specific effector T cells in the dLN and their subsequent brain infiltration. These events are all crucial set-points in the cancer-immunity cycle [47]. The combination treatment further increased myeloid PD-L1 expression in the dLN, whereas PD-L2 expression remained unaltered [59], supporting the in vitro observation that PD-L2 presumably plays no major role in GBM-mediated immunosuppression [30]. In conclusion, the individual potentials of both therapeutic arms become synergistically unlocked via a synchronized modulation of antigen presentation in the myeloid department.

#### Vaccination

Vaccination with glioma-associated antigen (GAA)-loaded DC or GAA peptides represents a popular immunotherapeutic strategy. Both in prophylactic and therapeutic settings, poly-ICLC significantly increased long-term survival of GL261-bearing mice treated with GAA vaccines [39, 60]. This was characterized by enhanced GAA-specific TIL, due to upregulated CXCL10 secretion in the TME, and development of immune memory [39, 60]. The combination strategy generates an IFN-γ-driven positive feedback loop (by lymphocytes) of the type I IFN polarization process (by tumor and myeloid cells) in the TME, causing a CXCL10 burst at the tumor site which is crucial for the recruitment of GAA-specific T cells [60]. Hence, poly-ICLC is critical for efficacious GBM vaccination. An addition of anti-CCL2 to inhibit tumor infiltration of immunosuppressive cells did not significantly improve survival further [61]. Interestingly, the distance from the tumor to the vaccination site appears correlated to therapeutic efficacy. The GBM tumor was able to prevent or suppress CTL priming when vaccinated in the cervical dLN or axillary LN, respectively, whereas a tumoricidal CTL response was only robustly develop by poly-ICLC-adjuvanted vaccination in the inguinal LN [62]. In summary, both poly-ICLC and vaccination site are critical determinants in GBM vaccination success.

#### Other combinations

In the nineties, the combination of poly(I:C) with cycloheximide resulted in IFN-β production and tumor growth inhibition in GBM-bearing nude mice, which was further strengthened by targeting poly(I:C) to the cytosolic receptors via liposome entrapment [28]. Recently, the combination of poly(I:C) with a small molecule antagonist of the inhibitor of apoptosis (LCL161), also called a Smac mimetic compound, increased the 17% survival rate for each monotherapy to 86% in combination in CT-2A-bearing mice [58]. This synergy was based on the sensitizing effect of LCL161 to TNF-α-induced tumor cell death in conjunction to induction of TNF-α secretion by poly(I:C). In addition, Smac mimetics block the growth-promoting activities of TNF-α, rendering this an intriguing combination option [58]. Last year, Yin et al. reported the intranasal delivery of poly(I:C) bound to gold nanoparticles, as a means to bypass the BBB and target the tumor cells. This extended the survival of mice treated with the conventional drug TMZ. The authors postulated that the combination was required to induce a coordinated release of IFN-β and killing of tumor cells by poly(I:C) and TMZ, respectively, resulting in immunogenic cell death (ICD) [40]. Given that TMZ remains SOC for newly-diagnosed GBM patients, this observation further encourages the inclusion of poly(I:C), as part of an immunotherapy, in the current treatment regimen for GBM.

### Clinical studies with poly-ICLC in GBM

In the earlier clinical GBM studies poly-ICLC was used as stand-alone treatment or in combination with (chemo)radiation, but lately is has mostly been used as a GBM vaccine adjuvant. Below we discuss the reported clinical findings in humans. Our methodological search found thirteen peer-reviewed reports on eleven clinical studies, as shown in Table 1: four non-vaccination trials [63–66] and seven vaccination trials as adjuvant to DC (three) or peptides (four) [67–75]. Newly-diagnosed and/or recurrent GBM were enrolled in four phase I, four phase I/II and three phase II trials. These studies enrolled a total of 323 patients, of which 227 suffered from GBM and another 21 from unspecified high-grade glioma (HGG). Only one study had a control arm for poly-ICLC [74]. Table 2 provides an overview of five currently ongoing trials.

**Table 1:** Overview of peer-reviewed published results of clinical GBM trials involving poly-ICLC

Author Year Trial ID	Diagnosis (n^a^) Phase	Treatment following diagnosis	Poly-ICLC	Safety	Immunomonitoring	Clinical effects For GBM patients unless specified otherwise
***Poly-ICLC in non-vaccination trials***						
*Salazar* *1996* *n.r.*	ND (18/38) & Rec (9^b^/38) Pilot (I/II)	/ or CT	10-50 μg/kg IM 1-3x/w alone or concurrent	Very well tolerated, = QoL	Serum: ↗↗ IFN (4/15), variable neopterin and IL-2, = TNF-α and IL-6	*ND GBM*: mOS 11mo (1x/w), 19mo (2-3x/w); 1 CR, 2 PR, 6 SD, 8 PD; 8/12 ≥ SD if 2-3x/w *recGBM*: mOS 15mo-19mo (w-w/o CT)
*Butowski* *2009* *NCT00052715*	ND (30) II	Surgery + RT	20 μg/kg IM 3x/w, concurrent + adjuvant	Very well tolerated, = QoL 15 AE3 + 1 AE4	n.r.	Study vs ctrl (RT + nonTMZ-CT) vs ctrl (RT): 12mo OS: 69.4 vs 57 vs 35% mOS: 15.0 vs 13.1 vs 9.2mo Study: 30 and 5% 6mo and 1y PFS, 18w TTP
*Rosenfeld* *2010* *NCT00262730*	ND (97) II	Surgery + Stupp	20 μg/kg IM 3x/w, concurrent (w2–8/cycle TMZ^adj^)	Attributed to poly-ICLC: 1 AE3 + 0/15 AE4 (−/+ TMZ) + 0/2 deaths	n.r.	Entire cohort vs 18-70y old cohort vs Stupp: mOS 17.2mo vs 18.3mo vs 14.6mo 12mo OS 73.2 vs 79.5 vs 61.1% 18mo OS 47.4 vs 51.8 vs 39.4% 24mo OS 29.9 vs 32.5 vs 26.5% Failure (death) HR 0.46 vs n.d. vs 0.63
*Hartman* *2014* *n.r.*	Rec, pediatric (12^b^/32) II	/	20 μg/kg IM 2x/w in 4w-cycles (until 2y)	Very well tolerated 16 AE3 + 3 AE4 (entire cohort)	n.r.	RecHGG: 1 PR (recAA), 1 SD, 1 response; 3/12 response rate overall
***Poly-ICLC as cancer vaccine adjuvant***						
*Okada* *2011* *NTC00766753*	Rec (13/22) I/II	GAA-loaded αDC1, IN Initiation (1-12w): 1x/2w Booster 1 (13-29w): 1x/4w Booster 2 (30w-3y): 1x/3mo	20 μg/kg IM, concurrent Initiation: 2x/w (w1–8) Booster 1: 2x/w Booster 2: 1x/w	≤ AE2	8/12 functional GAA-specific CD8^+^ T-cell response ↗ Type 1 cytokines & chemokines	1 CR, 1 PR, 9 SD mOS 12 mo mTTP 4mo 12mo PFS 30.8%
*Prins* *2011* *NTC00068510*	ND & Rec (11/23) I	ND: Surgery + Stupp; Rec: Surgery + Stupp + Surgery Autologous tumor lysate-pulsed DC, ID Initiation: 3x biweekly, before TMZ^adj^ Booster: 1x/3mo in between TMZ^adj^ cycles	/	≤ AE2	↗ Serum Th1 cytokines, ↗ Th1/Th2 cytokine ratio = Tregs; ↗ CD8^+^ TIL in mesenchymal group	↗ OS with DC vaccine vs controls in mesenchymal group; = OS in proneural group mOS 17.3 (DC only) vs ≥ 22.3mo (DC + poly-ICLC)
	ND & Rec (3/23) I		20 μg/kg IM, concurrent (boosters)	≤ AE2	Log-fold ↗ serum Th1 cytokines	
*Pollack* *2014* *NCT01130077*	ND, pediatric (5/26) Pilot	Surgery + Stupp (no TMZ^adj^) GAA in Montanide ISA-51, SC Initiation: 8x, every 3w Booster: every 6w until 2y	30 μg/kg IM, concurrent (vaccine)	≤ AE2	ELISPOT (3/5): 3/3 response to 2/3 GAA, 2/3 to GAA + Tet	2 CR, 1 PR, 2 SD mOS ≥14.7mo
*Pollack* *2016* *NCT01130077*	Rec, pediatric (6/12) Pilot	Various regimens GAA in Montanide ISA-51, SC Initiation: 8x, every 3w Booster: every 6w until 2y	30 μg/kg IM, concurrent (vaccine)	≤ AE2	ELISPOT (5/6): 5/5 response to ≥1 GAA, 2/5 to all GAA + Tet, 3/5 only to 1 GAA	1 PR, 2 SD, 3 PD mOS 14.25mo mPFS 1.8mo
*Hilf* *2018* *NTC02149225*	ND (15) I	Surgery + Stupp Personalized GAA vaccines APVAC1 + APVAC2, ID (+GM-CSF) during TMZ^adj^ APVAC1: pre-manufactured unmutated antigens, 11x APVAC2: neo-epitopes, 8x	1.5 mg SC, concurrent (vaccine)	6 > AE2 due to APVAC1/2, poly-ICLC and/or GM-CSF	*APVAC1*: 12/13 persistent response to ≥1 APVAC1-GAA, ↗ % AVPAC1-reactive memory T cells; ↗ PD-1; 9/13 Th1 CD4^+^ T cell response to ≥1 pan-DR antigens *APVAC2:* 8/10 neo-GAA-specific, CD4^+^ T cell response to APVAC2; 11/13 mutated APVAC2-GAA induced CD4^+^ T cell response (Th1 and multifunctional); no isolated CD8^+^ T-cell response	mOS 29.0mo mPFS 14.2mo
*Keskin* *2019* *NCT02287428*	ND, MGMT-unmethylated (8) I/Ib	Surgery + Stupp (no TMZ) Multi-epitope personalized neo-GAA vaccine Priming: 5x Booster: 2x	0.5 mg SC, admixed (vaccine)	≤ AE2	If no DEX during vaccination priming (2/8): IFN-γ response by polyfunctional neo-GAA-reactive T cells Generation of GAA-experienced memory T cells At relapse: ↗ CD8^+^ TIL, ↘ Treg. GAA-specific TCR-α and -β clonoypes found ↗ CD8^+^PD-1^+^ T cells	All patients died from PD mOS 16.8mo mPFS 7.6mo
*Migliorini* *2019* *NCT01920191*	ND (6) I/II	Surgery + Stupp IMA950 multi-GAA vaccine, ID Before and between TMZ^adj^ cycles	1.5 mg IM, concurrent (vaccine)	1 AE3 + 1 AE4 due to vaccine and/or poly-ICLC	*Concurrent*: ↘ specific T-cell response, ↘ immunization efficacy *Admixed (SC/IM):* ↗↗↗ immunization efficacy; ↗↗↗ mono & multiple GAA-specific CD4^+^ and CD8^+^ T-cell responses; durable type I cytokine secretion from 1^st^ vaccination GAA-specific TIL in 0/5 tumor samples after vaccination *GBM cohort:* 62.5 and 31.3% CD8^+^ T-cell response to 1 and > 1 GAA	*Concurrent*: 2 SD, 4 PD *Admixed, SC*: 1 CR, 5 PD *Admixed, IM*: 2 PR, 1 SD, 1 PD *GBM cohort*: mOS 19mo mPFS 9.5mo 6mo PFS 69% 9mo PFS 56%
	ND (6/7) I/II		1.5 mg SC, admixed (vaccine)			
	ND (4/6) I/II		1.5 mg IM, admixed (vaccine)			
*Boydell* *2019* *NCT01920191*	Rec (35/40) I/II post-hoc	Surgery + Stupp + Surgery 2^nd^ line: bevacizumab, 10 mg/kg IV every 2–3 weeks	/	n.r.	n.r.	OS and PFS = between cohorts
	Rec (14/16) I/II post-hoc	Surgery + Stupp + IMA950 vaccine + Surgery 2^nd^ line: bevacizumab, 10 mg/kg IV every 2–3 weeks	1.5 mg, admixed (IMA950 vaccine)			
*Wang* *2020* *NCT02709616* *NCT02808364*	ND GBM (3/5) Rec GBM (2/5) I	ND: Surgery + Stupp Rec: Surgery + Stupp + surgery GAA-loaded DC, ID/IV (+ CPM + imiquimod) 1 week after surgey	50 μg/kg poly(I:C) IM, concurrent (every 2 days for 2 weeks per vaccination)	≤ AE2	↗ GAA-specific CD4^+^ and CD8^+^ T cells in 3/3	mOS 19mo (vs 11mo)

*AA* astrocytic astrocytoma; *AE#* grade # adverse event; *CPM* cyclophosphamide; *CR* complete response; *Ctrl* historic control population; *DC* dendritic cell; *DEX* dexamethasone; *GAA* glioma-associated antigen; *GBM* glioblastoma; *GM-CSF* granulocyte-macrophage stimulatory factor; *HGG* high-grade glioma; *HR* hazard ratio; *ID* intradermal; *IFN* interferon; *IL-2/6* interleukin 2/6; *IM* intramuscular; *IN* intranodal; *MGMT* O-6-methylguanine-DNA methyltransferase; *n* number of patients; *ND* newly-diagnosed; *n.r.* not reported; *mo* months; *mOS* median overall survival; *PD* progressive disease; *PD-1* programmed cell death 1; *mPFS* median progression-free survival; *Poly(I:C)* polyinosinic:polycytidylic acid; *Poly-ICLC* polyinosinic:polycytidylic acid stabilized with carboxymethylcellulose and poly-L-lysine; *PR* partial response; *QoL* quality of life; *Rec* recurrent; *RT* radiotherapy; *SC* subcutaneous; *SD* stable disease; *Stupp* Stupp protocol of RT-CT/CT [1]; *TCR* T-cell receptor; *Tet* tetanus toxoid; *TMZ*^*(adj)*^, temozolomide (in adjuvant setting of Stupp protocol); *Treg* regulatory T cell; *mTTP* median time-to-progression; *w-w/o* with-without; ^a^, fractures denotes number of GBM patients in total cohort; ^b^, unspecified mix of HGG including GBM

**Table 2:** Overview of ongoing clinical glioblastoma trials involving poly-ICLC

Trial ID Phase	Diagnosis (n^**estim**^)	Treatment following diagnosis	Poly-ICLC
***Ongoing trials***			
*NCT03422094* I	ND, unmethylated (30)	Surgery + Stupp Investigational procedure during TMZ^adj^: NeoVax, SC (4x priming in cycle 1 + 1 booster/cycle), **+**	1.5 mg SC, admixed (vaccine)
		Nivolumab, 480 mg IV (start at progression)	
		Nivolumab, 480 mg IV (start with cycle 2)	
		Nivolumab, 480 mg IV (start with cycle 1)	
		Nivolumab, 480 mg IV (start with cycle 3) Ipilimumab, 1 mg/kg IV (cycle 1)	
		Nivolumab, 3 mg/kg IV (2x/cycle) Ipilimumab, 1 mg/kg IV (every 6 weeks)	
*NCT04201873* I	Rec (40)	Surgery + ALT-DC vaccine ID	IM, with vaccine
		Surgery + ALT-DC vaccine ID Pembrolizumab, IV, neo-adjuvant to surgery	
*NCT03223103* Ia/Ib	ND (20)	Surgery + Stupp Investigational procedure during TMZ^adj^:	100 μg/dose, with vaccine
		Mutation-derived GAA-based personalized vaccine Tumor-treating fields (continuous)	
*NCT03665545* I/II	Rec (24)	IMA950 vaccine, SC	SC, admixed (vaccine)
		IMA950 vaccine, SC Pembrolizumab, IV	
*NCT01204684* II	ND^a^ & Rec^a^ (30)	Autologous tumor lysate-pulsed DC vaccine	/
		Autologous tumor lysate-pulsed DC vaccine 0.2% resiquimod, concurrent (vaccine)	/
		Autologous tumor lysate-pulsed DC vaccine	Concurrent (vaccine)
***Completed trial, but no peer-reviewed report yet***^c^			
*NCT02078648* II	Rec (28^b^)	SL-701 vaccine, biweekly 10 mg/kg bevacizumab, IV, concurrent (vaccine)	IM, concurrent (vaccine)

*DC* dendritic cell; *GAA* glioma-associated antigen; *IM* intramuscular; *IV* intravenous; *n*^*estim*^ estimated enrollment; *ND* newly-diagnosed; *Poly-ICLC* polyinosinic:polycytidylic acid stabilized with carboxymethylcellulose and poly-L-lysine; *Rec* recurrent; *SC* subcutaneous; *Stupp* Stupp protocol of radiotherapy with concurrent and adjuvant chemotherapy [1]; *TMZ*^*adj*^ temozolomide in adjuvant setting of Stupp protocol; ^a^, high-grade glioma including glioblastoma; ^b^, accrued number of patients; ^c^, conference abstract available [76]

#### Safety and administration route

In the first pilot study in GBM, long-term low-dose intramuscular (IM) administration of poly-ICLC demonstrated safety within the HGG participants [66]. Such dosing scheme alleviated toxicities observed earlier in other indications that employed intravenous injection [12]. Most clinical GBM studies retained the IM route, although the three more recent trials administered it subcutaneously (SC). One trial comparing both administration routes could not determine the best choice, as both elicited multi-GAA immunity [70]. In total 80 grade 3 AE and 23 grade 4 AE were possibly due to poly-ICLC, although 16 AE3 and 3 AE4 were possibly observed in non-GBM patients [64]. One AE3 rash could be definitely attributed to poly-ICLC [65]. In the study that controlled for poly-ICLC adjuvant, no increased toxicities were observed apart from mild transient fevers upon vaccination in 2/3 of the patients [74]. The most common AE included injection site reactions and flu-like symptoms, but in trials with TMZ, leukopenia was also described. Overall, poly-ICLC was well-tolerated by GBM patients without a clear negative impact on the quality of life.

#### Immunological effects

Due to its immunostimulatory effects, poly-ICLC has mostly been employed as a cancer vaccine adjuvant in order to mature DC. Enhanced antigen presentation and T-cell priming then improves the generation of antigen-specific immune responses [12]. In the poly-ICLC-adjuvanted GBM vaccination studies, GAA-specific CD4^+^ and/or CD8^+^ T-cell responses were observed in 43/57 patients (75.4%; range 40.0–100.0%), of which 69.8% showed T-cell reactivity against multiple GAA [68–73, 75]. Notably, Migliorini et al. observed that admixing poly(I:C) with the IMA950 peptide vaccine drastically enabled the induction of specific T-cell responses compared to concomitant poly-ICLC by augmenting the immunization efficacy (16.7 to 92.3%) [70]. Nonetheless, other studies employing concomitant administration observed specific T-cell responses at higher rates as well [68, 72, 73, 75]. Two studies reported the generation of polyfunctional T cells, the induction/upregulation of antigen-reactive Tmem and the moderate or high expression of PD-1 [68, 69]. Despite their systemic presence, one trial did not find vaccine-specific TIL in 5/5 samples at relapse after vaccination [70]. On the other hand, another study observed increased GAA-specific CD8^+^ TIL, with elevated PD-1^+^ at relapse following vaccination, suggesting prior activation, and only few Treg [69]. Interestingly, immunological responses were absent in patients who were on dexamethasone during the priming phase of the vaccination [69]. This should be taken into account during future trial design, since dexamethasone is frequently prescribed to alleviate cerebral edema in GBM patients. The sole trial with a control arm for poly-ICLC did not report on GAA-specific T-cell responses but instead described a log-fold increase in Th1 cytokine secretion when poly-ICLC was concomitantly administered during boosters [74]. Likewise, other trials reported increased Th1 cytokine secretion or production, which Migliorini et al. proved durable from the first vaccination onwards in the admixed formulation [70, 71]. Only one non-vaccination study reported on immunomonitoring, with few serum cytokine changes, most notably an IFN increase [66]. No studies could relate the observed immunological effects to preliminary clinical response nor endogenous GAA expression [66, 70, 75]. Overall, the lack of immunomonitoring in non-vaccination GBM trials prevents the thorough evaluation of immunological responses in these settings. In contrast, poly-ICLC-adjuvanted vaccination has proven to be immunogenic in GBM patients, which is supported by data of randomized clinical trials in other indications [12].

#### Clinical effects

Only three studies were phase II, and notably, none of them considered a vaccination trial [63–65]. The other trials were less qualified to report on clinical effects, although the phase I/II studies already render a glimpse into potential clinical benefits. Overall, the following objective response rates (ORR) were observed in a combined total of 63 attestable GBM patients who received poly-ICLC: five complete responses, seven partial responses, 22 stable diseases and 29 progressive diseases [66, 69–73]. This culminates in a disease control rate (DCR) of 46.9%. However, since ORR and DCR are subjective to time, we focus on more robust parameters, e.g. overall survival (OS), progression-free survival (PFS) and time-to-progression (TTP). The landmark 2005 phase III EORTC trial on TMZ will serve as a frame of reference (1).

The one study prior to the landmark TMZ trial investigated poly-ICLC as stand-alone or in combination with concurrent chemotherapy. For newly-diagnosed GBM patients, mOS was 11 months with a single weekly injection, but rose to 19 months when 2–3 injections per week were administered [66]. Remarkably, this is longer than the contemporary reference of 14.6 months. Of note, this considered a phase I/II trial and hence, underpowered for conclusive assessment of therapeutic efficacy. Also noteworthy, the few recurrent GBM patients survived for 15 or 19 months, depending on the presence/absence of concurrent chemotherapy [66]. Redefinition of the SOC to include TMZ following the EORTC trial prematurely terminated the exploration of poly-ICLC concurrent plus adjuvant to radiotherapy in newly-diagnosed GBM patients. OS was similar to the EORTC trial, but the time to progression was shorter, although results were improved compared to radiotherapy alone [63]. As such, it is curious whether poly-ICLC could have made it to phase III and the SOC, if the EORTC trial would not have been. Thereafter, poly-ICLC during the adjuvant TMZ maintenance phase of the Stupp protocol showed improved OS compared to the landmark TMZ trial, even more so when only the 18–70-year old cohort was considered (mOS 17.2 and 18.3 months, respectively). Consequently, the total group showed a reduced hazard ratio for death (0.46 vs 0.63) [65]. Importantly, Rosenfeld et al. acknowledged the presence of confounding factors in their comparison with the EORTC trial, such as the smaller scale of their multi-institutional network, i.e. 6 vs > 80, the latter for which it is nearly impossible to apply highly similar treatment approaches [65]. Ultimately, with the rise of immunotherapy for brain cancer, the focus with poly-ICLC shifted toward adjuvanting vaccines.

The vaccination studies look promising, keeping in mind they are underpowered. For instance, the studies reported by Okada et al. and Pollack et al. achieved similar results as the TMZ landmark trial but were performed on recurrent GBM, which has an even worse prognosis [71, 72]. In addition, Pollack reported a mOS of 25.1 months with poly-ICLC-adjuvanted vaccination in newly-diagnosed pediatric GBM patients [73]. In a trial enrolling both newly-diagnosed/recurrent GBM, mOS rose from 17.3 to 22.3 months when poly-ICLC was added [74]. While this hints at the potential for poly-ICLC, the authors emphasized its contribution was unclear since it was only administered with the booster vaccines [74]. These investigators are currently running a follow-up phase II trial (NCT01204684; Table 2). The recent poly-ICLC-adjuvanted multipeptide vaccination studies also reported promising clinical effects on small sample sizes, ranging from 16.8–29.0 months mOS and 7.6–14.2 months mPFS [68–70]. A post-hoc exploration of the poly-ICLC-adjuvanted IMA950 vaccine regarding increased sensitivity to bevacizumab after relapse concluded a negative result, although the authors suggest a concurrent use may unlock potential synergy in this immune/anti-angiogenic combination [67]. This concurrent strategy was investigated in a completed phase II trial (NCT02078648; Table 2). While awaiting the definite report, the disclosed preliminary findings describe major responses and a promising survival curve [76].

In summary, although preliminary findings indicate beneficial responses when combined with the SOC, the future of poly-ICLC in GBM lies within immunotherapy regimens. Nonetheless, despite evidence of immunological activity in poly-ICLC-adjuvanted vaccination studies, additional phase ≥II studies involving more patients are warranted to reliably evaluate the promising preliminary clinical observations. Presumably, combination with other treatments such as ICB is required to adequately potentiate the immunotherapeutic effect.

#### Ongoing clinical studies

Table 2 presents the five currently ongoing clinical trials for GBM patients with poly-ICLC as an investigational drug. Notably, all trials consider vaccination studies. Three studies investigate the addition of ICB. Another trial assesses the addition of poly-ICLC-adjuvanted vaccines to tumor-treating fields, the latest approved, though controversial, modality for GBM. The last clinical trial considers a phase II study following the phase I study of Prins et al. discussed above [74].

## Discussion

Safety is the primary concern for any drug. Unlike treatment of hepatocellular and pharyngeal carcinoma cell lines [25, 26], poly(I:C)-induced migration or invasion of GBM cells is not reported. In fact, whereas no pro-tumorigenic effects on GBM cell health have been reported, poly(I:C)-induced cytotoxic outcomes have been described numerously, which is in line with the innate defense mechanism of virus-infected cells against viral spreading [24]. Moreover, in vivo safety and tolerability has been evidenced. Indeed, poly(I:C)-related safety issues from the early days have been dealt with [12], as evidenced by 57 ongoing (of 119 total registered) clinical trials (clinicaltrials.gov). Nonetheless, more definitive data on migration and invasion of GBM cells following poly(I:C) treatment could provide closure of this lingering issue.

The formulation of poly(I:C) requires more research, given that this determines which receptors are primarily targeted. While TLR-3 and the cytoplasmic receptors share overlapping signaling cascades, their differences can modulate the height or presence/absence of a certain response. In this context, preclinical data suggest that increased targeting of the cytosolic receptors might increase tumorlysis [28, 31, 54, 55]. Clinical application of poly(I:C) in GBM has been limited to poly-ICLC (Hiltonol, Oncovir). Recently, a nanoplexed form of poly(I:C) coupled to polyethylenimine (BO-112, Bioncotech Pharmaceuticals), has entered the clinical phase for solid tumors, where it will be combined with anti-PD-1 (NCT02828098). Interestingly, BO-112 was found superior to poly(I:C) in preclinical non-GBM tumor models following intratumoral administration [77]. For the purpose of accommodating GBM patients, a comparison between BO-112 and poly-ICLC in GBM is warranted. In addition, novel alternatives are being developed, e.g. a short dsRNA monoclonal antibody (TL-532, Tollys) and DNA-capped dsRNA (ARNAX, Hikkaido University). ARNAX performed similarly to poly(I:C) in wild-type and tumor-bearing mice, though with minimal cytokine secretion to enhance its tolerability [78].

**Fig. 4:**
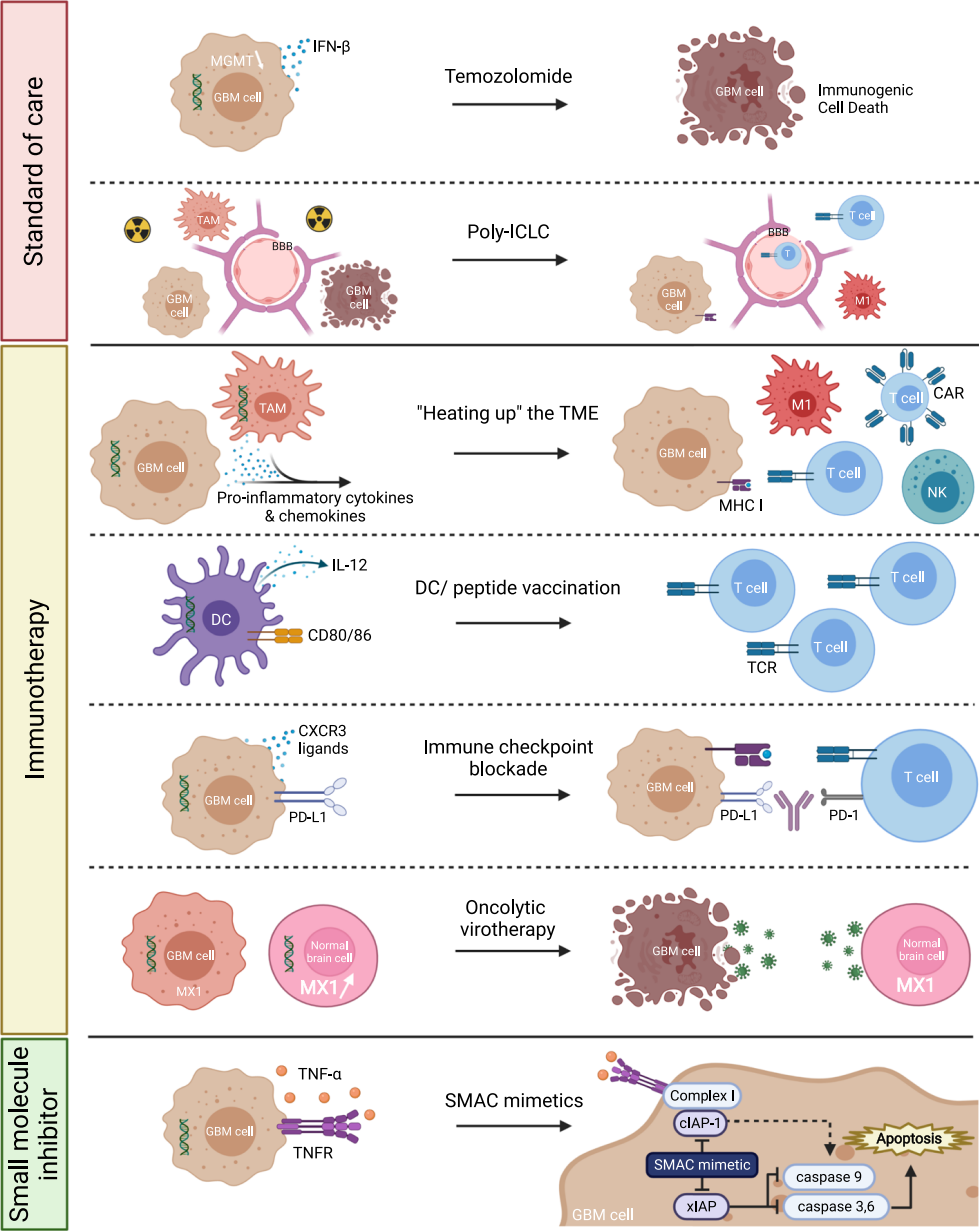
Poly-ICLC driven therapeutic combinations options in GBM. Poly-ICLC possesses abilities to propel standards of care modalities, but in particular immunotherapy. Given the interplay between poly-ICLC and the different potential therapeutic partners, we postulate to combining more than two components outside of the standard of care will be required to, and bears promising potential to, invigorate treatment of GBM patients. Green helices represent double-stranded poly-ICLC. BBB, blood-brain barrier; CAR, chimeric antigen receptor; cIAP-1, cellular inhibitor of apoptosis protein 1; CXCR3, C-X-C motif chemokine receptor 3; DC, dendritic cell; GBM, glioblastoma; IFN, interferon; IL-12, interleukin-12; M1, M1-polarized macrophage; MGMT, O^6^-methylguanine-DNA methyltransferase; MHC, major histocompatibility complex; MX1, MC dynamin-line GTPase 1; NK, natural killer cell; PD-(L)1, programmed death 1 (ligand 1); poly-ICLC, polyinosinic:polycytidylic acid stabilized with carboxymethylcellulose and poly-L-lysine; SMAC, second mitochondrial activator of caspases; TAM, tumor-associated macrophage; TCR, T-cell receptor; TME, tumor microenvironment; TNF(R), TNF, tumor necrosis factor (receptor); xIAP, elevated x-linked inhibitor of apoptosis protein

Poly(I:C) activates immune cells and it seems that GBM cells respond similarly, secreting pro-inflammatory and lymphocyte-attractive cytokines. Such an immunostimulatory environment is required to render GBM susceptible to immunotherapy. Notably, the pro-inflammatory mesenchymal GBM subtype is particularly immunogenic and more susceptible to immunomanipulation [74, 79]. Moreover, GBM is considered a cold tumor, lacking high numbers of non-exhausted TIL and an adequate antigen presenting machinery [4]. Preclinical data suggest poly(I:C) could aid in overcoming these hurdles, as visualized in Fig. 4. Antigen presentation could be targeted by poly(I:C)-mediated DC activation. Concerning the immunological temperature of GBM, poly(I:C)-mediated Th1 cytokine and chemokine secretion has been clinically observed [70, 71, 74]. Importantly, such Th1 secretome – driven by CXCR3 ligands – was preclinically demonstrated as critical for cytotoxic lymphocyte recruitment enabling the efficacy of glioma vaccines as well as ICB in solid tumors [60, 80]. These observations are further supported by the poly(I:C)-mediated conversion of immune-cold to immune-hot lesions in melanoma [81]. In addition, systemic poly(I:C) enables brain trafficking of α4-integrin-deficient lymphocytes [82]. This could also open up avenues for stimulating tumor influx of CAR lymphocyte products. While poly-ICLC did not render GBM immune-hot in the clinical vaccination studies, increased numbers of GAA-specific T cells were detected [70]. Moreover, an unbiased systems approach in two solid tumor mouse models identified poly(I:C) as one of three critical compounds to convert the TME susceptible to ICB by upregulation signal of transducer and activator of transcription 1 and TLR-3 signaling [83]. Rational combination strategies and modulation of administration/formulation may further close the gap between these two TME immunological phenotypes. An intriguing option to accelerate this phenomenon could be to transiently deliver poly-ICLC intratumorally following surgical resection via injection, lavage or micropumps, although the effect on healthy brain should be cautiously monitored.

Both vaccination and ICB are yet to deliver on their beneficial promises in GBM treatment, thus emphasizing the need for more rational strategies. Pre−/clinical evidence points towards a promising therapeutic triangle between poly(I:C), vaccination and ICB in GBM: (i) poly-ICLC-adjuvanted vaccination is capable of generating GAA-reactive PD-1^+^ T cells [68–71]; (ii) poly(I:C) is a strong maturation signal for DC antigen presentation [12]; and (iii) poly(I:C) has preclinically shown to synergize with ICB in GBM, dependent on antigen-presenting DC [30, 59]. Indeed, ICB not only alleviates inhibitory signaling in the TME, but also during DC-mediated T-cell priming in the dLN [84]. The field is evolving towards this triangle, as evidenced by fifteen ongoing trials combining these three therapeutic arms, of which two studies in GBM. Cross-presentation could further be potentiated by the addition of fms-like tyrosine kinase 3 ligand, or Flt3L [85]. In addition, the treatment scheme should be carefully considered. Recently, neo-adjuvant anti-PD-1 treatment was reported to double the mOS compared to the adjuvant setting in a small cohort recurrent GBM patients [86]. While vaccination in a neo-adjuvant setting is less feasible, administering poly-ICLC earlier in combination with ICB could render greater development of immunity. This triangle could be further potentiated by induction of ICD, despite limited clinical evidence of ICD in GBM [4]. The novel BO-112 appears to be a strong ICD inducer, contrary to poly(I:C) [77]. This may unlock closed avenues in the treatment strategy.

Smac mimetics lead to tumor cell apoptosis when combined with agents that induce IL-1β, IFN-β, TNF-α and TRAIL [58]. Since poly(I:C) induces such secretion by tumor and stromal cells [23, 30], its recent preclinical synergistic pairing with Smac mimetics in GBM holds an intriguing promise that warrants further investigation [58]. Moreover, Smac mimetics also synergize with oncolytic viruses and ICB, a duo which, in combination, also has demonstrated preclinical success [58, 87]. Hence, one could envision a multimodal treatment strategy wherein poly(I:C) (i) guides tumor cell apoptosis, (ii) potentiates anti-tumor immunity, and (iii) confers protection for normal human brain cells [30, 42, 58].

While we have focused on the GBM cells in the in vitro studies, brain tumor-infiltrating microglia and macrophages (BTIM) should not be neglected. This cell population presents an obstinate problem in GBM given their prevalence and GBM-supporting/immunosuppressive phenotype [88]. Indeed, depolarization of pro-tumorigenic BTIM via inhibition of CSF-1R resulted in GBM regression and increased survival [89]. Alternatively, poly(I:C) converted pGBM-derived BTIM towards an oncotoxic, phagocytic and locomostatic profile against pGBM [23], similar to observations in a lung tumor model [90]. In addition, poly(I:C) leads to the secretion of macrophage-derived CXCR3 ligands that have been identified as fundamental for therapeutic efficacy of ICB [21, 22, 80]. Hence, adding poly(I:C) to an immunotherapeutic regimen may also tackle the largest immunosuppressive cell population in the TME of GBM and redirect them to lymphocyte attractors, thus further empowering its potential to enhance the cancer-immunity cycle. Notably, BTIM pre-stimulation with poly(I:C) was required to overcome GBM-induced suppression [23], which rationalizes the application of poly(I:C) after surgical tumor debulking. Of course, poly(I:C)-induced activation of antigen-presenting cells, particularly DC [13], should not be overlooked and is a crucial part of its mode of action.

Until immunotherapy breaks through as an approved therapeutic option for GBM, the SOC still consists of chemoradiotherapy. Responsiveness to TMZ is dictated by O^6^-methylguanine-DNA methyltransferase (MGMT) promotor methylation status [91]. IFN-β can sensitive GBM cells to TMZ by suppressing MGMT activity [92], although the SOC + IFN-β did not lead to a significant survival benefit in the phase II INTEGRA trial [93]. However, a recent murine orthotopic GBM study demonstrated a significant survival prolongation upon nasally administered brain-targeted delivery of poly(I:C) to circumvent the BBB [40]. Here, poly(I:C) produced endogenous IFN-β while attenuating MGMT, allowing TMZ to kill the GBM cells in an immunogenic manner [40]. Given that clinical studies with poly-ICLC in GBM have yielded modest results, a key to success may lie in bringing poly(I:C) to the tumor. Indeed, poly(I:C) in mouse models of other tumor types has been very effective when administered intratumorally [77, 81]. The other SOC component, radiotherapy, has been attributed to have immunogenic properties. Combination with poly(I:C) could be beneficial by stimulating the antigen-presenting cells and subsequent T-cell trafficking, facilitated by a radiation-induced increase in BBB permeability, in order to fully engage with radiation-induced ICD [94]. On the other hand, radiotherapy could also have immunosuppressive effects, including induction of M2 macrophages [95], which can be repolarized by poly(I:C) [23]. Intriguingly, despite interesting clinical effects in a phase II trial, poly-ICLC complimentary to the Stupp protocol was not further investigated [65], although its true value would lie in adjuvanting immunotherapy.

Although preclinical evidence regarding poly(I:C) in GBM treatment is very promising, the clinical effect is less pronounced. While immunological activity was observed in the vaccination studies, their statistical power preaches prudence regarding conclusions on clinical benefit. The incidence of GBM, a patient’s fitness and the number of ongoing GBM studies are limiting factors in achieving sufficient patient enrollment for large sample sizes. Indeed, GBM remains a rare disease that is more prevalent in elders, whose comorbidities may fit the inclusion criteria less [96], which hampers the estimated accrual of the 522 ongoing GBM clinical trials. Therefore, rational study designs based on solid preclinical foundations are crucial in guiding the most promising strategies towards the clinic. In order to increase the translation of preclinical results, higher-fidelity preclinical models and manipulations are warranted. In vitro work on “archaic” 2D-cultured GBM cell lines is being replaced by primary patient-derived tumor cell cultures, (brain-specific) 3D architectures and organoid models [30, 97–102], bringing the in vitro work closer to the reality in the clinic. Whereas GL261 remains the most commonly used animal model for GBM, genetically-engineered, humanized and immunotherapy-resistant mouse models [103, 104] will solidify the informational relevancy. Importantly, a full immune system and a complete display of all GBM hallmarks should be present in these animal models. In addition, adopting stratified models of GBM subtypes in preclinical research might guide patient selection in clinical trials, benefiting both patient and trial. Besides the constitution of in vivo models, the clinical procedures should be taken into account. In humans, GBM treatment is generally initiated by surgical resection. However, this procedure is seldomly carried out in mouse models. The recent neo-adjuvant ICB study has demonstrated the potential significance of the presence/absence of the primary tumor bulk in immunotherapeutic strategies [86], which could partly account for the poor clinical translation. In addition, the subsequent SOC procedures as well as symptomatic relief treatments are perpetually neglected, although they notoriously exert immunosuppressive effects, as recently demonstrated for dexamethasone [4, 69, 105]. Hence, to improve clinical translation, it will be imperative to not only create high-fidelity models, but also to align the preclinical therapeutic manipulations and experimental settings closely to the clinic. In the meantime, we eagerly await the results from the ongoing clinical trials.

## Conclusions

The value of poly(I:C) derivatives lies in their potential cytotoxic tumor effect and stimulation of anti-tumor immunity with a pivotal role for activating DC. By exerting these functions, it can interact at multiple stages of the cancer-immunity cycle [47]: (i) release antigens via (immunogenic) cytotoxicity, (ii) instigate antigen presentation by DC, (iii) aid T-cell priming by releasing IFN-α/β and other cytokines, (iv) stimulate T-cell recruitment via tumor- and macrophage-secreted chemokines, and (v) improve T-cell recognition of tumor cells via increased MHC expression. The accompanying increased PD-L1 expression should be a primary target to be leveraged for combination to further accelerate through this cycle. Hence, poly(I:C) derivatives could serve as central contributors in future immunotherapeutic strategies for GBM – primarily in an adjuvant role – of which multiple rational combinations hold promise. For these reasons, poly(I:C)-derivatives deserve substantial consideration as an adjuvant to boost immunotherapeutic clinical approaches for GBM.

## Data Availability

Not applicable.

## Abbreviations

AE: Adverse events
AP-1: Activator protein 1
BBB: Blood brain barrier
CAR: Chimeric antigen receptor
DC: Dendritic cell
DCR: Disease control rate
dLN: tumor-draining lymph node
GAA: Glioma-associated antigen
GBM: Glioblastoma
HGG: High-grade glioma
ICB: immune checkpoint blockade
ICD: Immunogenic cell death
IFN: Interferon
IKK: Inhibitor of NFκB kinase
IL: Interleukin
IM: Intramuscular
IRF: IFN regulatory factor
ISRE3: IFN-stimulated response element 3
MAVS: Mitochondrial antiviral signaling protein
MDA-5: Melanoma differentiation-associated gene 5
MHC: Major histocompatibility complex
(m)OS: (median) overall survival
NFκB: Nuclear factor κ-light-chain-enhancer of activated B cells
NK cells: Natural killer cells
ORR: Objective response rate
PD-L1/2: Programmed cell death 1/2 ligand
PEG: Pegylated
PFS: Progression-free survival
pGBM cells: primary human glioblastoma cells
pGSC: primary glioblastoma stem cells
Poly(I:C): Polyinosinic:polycytidylic acid
Poly-ICLC: Poly(I:C) stabilized with carboxymethylcellulose and poly-L-lysine
RIG-I: Retinoic acid-inducible gene I
RIP-1: Receptor-interacting serine/threonine-protein kinase 1
SC: Subcutaneous
SOC: Standard of care
TAK1: TGF-β activated kinase 1
TAM: Tumor-associated macrophage
TBK1: TRAF family member associated NFκB activator binding kinase 1
TGF-β: Transforming growth factor β
TICAM1: TLR adaptor molecule 1
TIL: Tumor-infiltrating lymphocytes
TLR-3: Toll-like receptor 3
TNF: Tumor necrosis factor
TME: Tumor microenvironment
Tmem: Memory T cells
TRAF: TNF receptor associated factor
Treg: Regulatory T cells
TTP: Time-to-progression
TMZ: Temozolomide
